# Au-Ag Bimetallic Nanoparticles for Surface-Enhanced Raman Scattering (SERS) Detection of Food Contaminants: A Review

**DOI:** 10.3390/foods14122109

**Published:** 2025-06-16

**Authors:** Pengpeng Yu, Chaoping Shen, Xifeng Yin, Junhui Cheng, Chao Liu, Ziting Yu

**Affiliations:** 1 School of Agricultural Engineering, Jiangsu University, Zhenjiang 212013, China; austchengjunhui@126.com; 2 School of Energy and Power Engineering, Jiangsu University, Zhenjiang 212013, China; shenchaoping0521@163.com; 3 Zhenjiang Agricultural Products Quality Inspection and Testing Center, Zhenjiang 212009, China; insnfeng@163.com; 4 Jurong Agricultural Product Quality and Safety Monitoring Center, Zhenjiang 212400, China; lc880724@163.com (C.L.); 18852881839@163.com (Z.Y.)

**Keywords:** Surface-enhanced Raman scattering, food contaminants, Au-Ag bimetallic nanoparticles, sensor

## Abstract

Food contaminants, including harmful microbes, pesticide residues, heavy metals and illegal additives, pose significant public health risks. While traditional detection methods are effective, they are often slow and require complex equipment, which limits their application in real-time monitoring and rapid response. Surface-enhanced Raman scattering (SERS) technology has gained widespread use in related research due to its hypersensitivity, non-destructibility and molecular fingerprinting capabilities. In recent years, Au-Ag bimetallic nanoparticles (Au-Ag BNPs) have emerged as novel SERS substrates, accelerating advancements in SERS detection technology. Au-Ag BNPs can be classified into Au-Ag alloys, Au-Ag core–shells and Au-Ag aggregates, among which the Au-Ag core–shell structure is more widely applied. This review discusses the types, synthesis methods and practical applications of Au-Ag BNPs in food contaminants. The study aims to provide valuable insights into the development of new Au-Ag BNPs and their effective use in detecting common food contaminants. Additionally, this paper explores the challenges and future prospects of SERS technology based on Au-Ag BNPs for pollutant detection, including the development of functional integrated substrates, advancements in intelligent algorithms and the creation of portable on-site detection platforms. These innovations are designed to streamline the detection process and offer guidance in selecting optimal sensing methods for the on-site detection of specific pollutants.

## 1. Introduction

Food safety is a crucial aspect of public health and social well-being, playing a vital role in maintaining and promoting human health. Food safety incidents have heightened public awareness of food safety concerns [1,2]. The primary pollutants responsible for food safety issues include pesticides, heavy metals, foodborne pathogenic bacteria, toxins and other harmful substances, all of which pose potential risks to human health [3]. Consumption of contaminated food can lead to a wide range of health problems, from mild gastrointestinal discomfort to severe, life-threatening diseases. Various methods are currently available for detecting harmful substances in food, such as molecular biology techniques [4], enzyme-linked immunosorbent assays [5], fluorescence sensors [6], colorimetry methods [7], electrochemical techniques [8], etc. While these detection methods offer high sensitivity, they often suffer from limitations such as complex procedures, long pretreatment times, high costs and susceptibility to interference.

Raman spectroscopy is a type of molecular vibrational spectroscopy that provides structural information about compounds [9]. However, its application is limited due to its low signal strength. The discovery of surface-enhanced Raman scattering (SERS) has significantly advanced the development and application of Raman spectroscopy [10]. SERS has unique advantages, such as a narrow peak shape, high sensitivity, fast response, no influence from water, and the ability to identify and quantify target compounds based on molecular fingerprints [11]. It is a rapid and non-destructive spectral analysis technique. It has been widely applied in fields such as early disease diagnosis [12,13], biological imaging [14], environmental monitoring [15] and food safety [16]. The enhancement mechanism of SERS technology for target molecules mainly includes electromagnetic enhancement based on electromagnetic fields and chemical enhancement based on charge transfer [17,18]. The localized surface plasmon resonance (LSPR) model based on the electromagnetic enhancement mechanism holds that when the incident laser irradiates the rough surface of the precious metal nanomaterial, the freely moving electrons on the surface of the precious metal generate plasmon resonance, thereby significantly enhancing the Raman signal [19]. The SERS response of a target molecule is primarily influenced by the enhancement effect of the SERS substrate. To achieve a higher SERS response, researchers have developed various substrates, including Au nanorods (AuNRs) [20], Ag nanoflowers [21], nanotriangles [22], Au nanostars [23], ZnO@ZIF-8 [24], WO_3-x_ nanowire/WSe_2_ heterostructures [25] and chiral carbon nanotube (CNT)/TiO_2_ hybrids [26].

Among the materials commonly used in SERS, precious metal nanomaterials, particularly Au and Ag, demonstrate exceptional performance due to their unique optical properties. First, their high surface area-to-volume ratio creates numerous hotspots where the electromagnetic field is significantly enhanced, thus amplifying the Raman signal [27]. Second, the tunability of their size, shape and composition allows for the optimization of SERS performance for specific applications. Currently, precious metal nanomaterials have evolved from single-component to multi-component forms. Au-Ag bimetallic nanoparticles (Au-Ag BNPs), such as Au-Ag core–shell nanoparticles, exhibit superior stability and SERS activity compared to single-metal nanoparticles, as strong local electromagnetic fields are generated through the coupling of different metallic materials [28].

**Figure 1:**
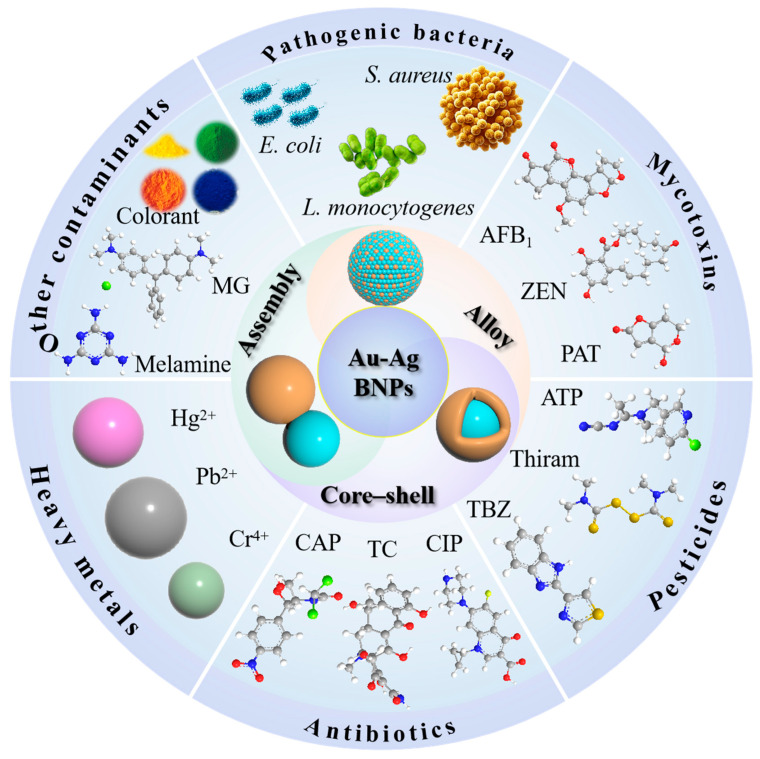
Schematic diagram of the types of SERS substrates based on Au-Ag bimetallic nanoparticles (Au-Ag BNPs) and their applications in food contaminants.

Extensive research has been conducted on the application of SERS in food safety detection, leading to the publication of several reviews with varying focuses. However, most of these reviews either address the application of specific target detection substances or primarily focus on the synthesis of a particular type of enhanced substrate [29,30]. In SERS technology, the enhancement effect of the substrate material plays a critical role. Bimetallic nanomaterials based on Au and Ag, exhibiting synergistic effects, typically demonstrate stronger SERS performance. Despite this, recent advancements in their synthesis and application have not been reported. This paper aims to highlight the significance of Au-Ag BNPs in the development of SERS technology by systematically summarizing their classification, synthesis and applications in detecting contaminants (Figure 1). Furthermore, this review anticipates the challenges and future prospects of using Au-Ag BNPs in SERS-based detection of harmful substances in food.

## 2. Types and Synthesis of Au-Ag BNPs

### 2.1. Types of Au-Ag BNPs

In recent years, precious metal nanoparticles have been developed to meet the growing demands of SERS detection. These nanoparticles can generally be classified into three categories based on their structural morphology: alloy, core–shell and aggregate types. The nanostructure of Au-Ag BNPs can provide a strong electromagnetic field and significantly enhance the Raman signal of the target molecules. Additionally, when combined with biomolecules such as aptamers and antibodies, these nanostructures offer multifunctionality, thereby broadening the scope of SERS applications [31].

Au-Ag alloy nanomaterials are formed by the uniform mixture of Au and Ag atoms at the atomic scale. In their structure, the two metal atoms are usually distributed in a disordered manner, forming nanoparticles with a single phase. The composition of the alloy can be continuously regulated by adjusting the Au/Ag ratio (such as Au_x_Ag_1−x_, x = 0.1~0.9). The particle morphology is predominantly spherical [32] but can also include cubic [33], octahedral [34] and other forms.

The core–shell structure is the most versatile and widely used. Core–shell nanostructures primarily exploit the strong electromagnetic field generated by the metal core to enhance the Raman signal of analytes located on or near the shell layer [35]. Core–shell structures are heterogeneous nanoparticles formed by layer-by-layer assembly of one metal (the core) and another metal (the shell), with a clear two-phase interface [36]. These structures can be classified into two types: “Au-core/Ag-shell” (Au@Ag) and “Ag-core/Au-shell” (Ag@Au). The core diameter typically ranges from 10 to 100 nanometers, while the shell thickness can be precisely controlled from a few nanometers to tens of nanometers. The core and shell are linked through lattice matching or surface ligands, generating core–shell interface effects such as charge transfer and stress regulation.

Au-Ag aggregates are multi-particle assemblies formed by the interconnection of individual Au/Ag nanoparticles (spherical, rod-shaped, star-shaped, etc.) through physical (such as electrostatic interaction and van der Waals forces) or chemical (such as ligand bridging and covalent bonds) actions, including dimer, trimer, chain, network or disordered aggregates [37]. A key characteristic of these aggregates is the presence of nanoscale gaps (typically 1~20 nm) between particles. These gaps create “hotspots” regions, where the intensity of the electromagnetic field increases exponentially, with enhancement factors ranging from 10^6^ to 10^12^. The structure of the aggregates can be finely tuned by controlling parameters such as particle size, morphology, spacing and connection methods (e.g., point contact and surface contact).

**Figure 2:**
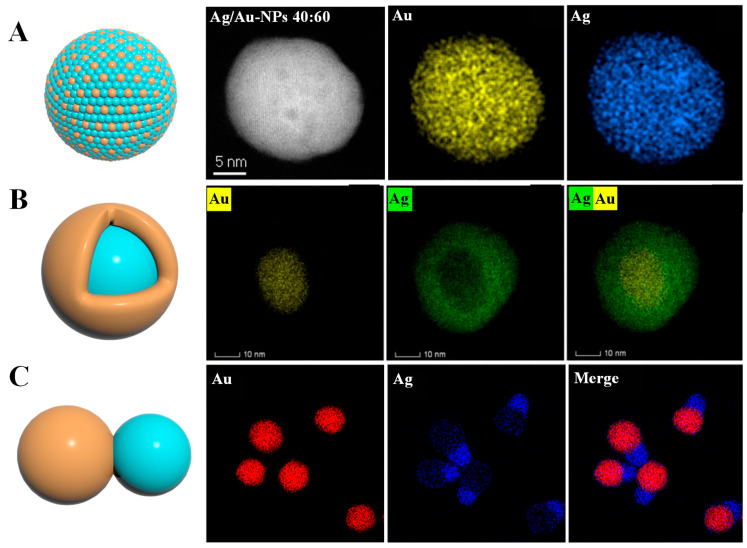
The types of Au-Ag BNPs and schematic diagrams of their model structures (orange and light blue respectively represent one of the Ag and Au elements). (**A**) Energy dispersive X-ray spectroscopy mapping of individual Ag/Au-NPs with atomic ratios (Ag:Au = 40:60) [38]. (**B**) The corresponding Au and Ag elemental mapping images of the Au@Ag NPs [39]. (**C**) Element mapping images of the Au-Ag JNPs [40].

To sum up, as shown in the model structure diagram in Figure 2, where orange represents the Ag element (or Au element) and the light blue represents the Au element (or Ag element), the Au and Ag elements in Au-Ag alloy nanomaterials are distributed in a disordered state on a single nanoparticle. Core–shell nanomaterials have Au and Ag elements distributed in a core–shell state as a whole on a single nanoparticle. Aggregates are equivalent to the coupling of Au nanoparticles and Ag nanoparticles, and the elements are distributed in two regions. The elemental mapping images representing different types of Au-Ag BNPs provided in the literature and presented in Figure 2 show that the distribution positions of the two elements (Au and Ag) constituting the nanoparticles are different.

### 2.2. Synthesis of Au-Ag BNPs

The effective synthesis of nanoparticles is crucial to their SERS enhancement. Consequently, understanding the common synthesis methods, particularly those for Au-Ag BNPs used in SERS detection, is essential. Currently, the synthesis of Au-Ag BNPs is typically classified into physical, chemical and biological methods. Physical methods offer advantages in the preparation of monodisperse and core–shell structures; however, their high costs and low yields limit large-scale application. Chemical methods are the most effective for regulating the structure of Au-Ag BNPs (such as alloys and core–shell configurations), and they are especially suitable for electrochemical and optical sensing. Biological methods hold promise in antibacterial and biomedical applications due to their environmental friendliness and biocompatibility, though challenges related to size control and repeatability remain to be addressed.

#### 2.2.1. Physical Synthesis Methods

Physical synthesis methods for nanoparticles typically involve breaking bulk materials into fine particles. These methods offer advantages such as a narrow particle size distribution and uniform performance. Laser ablation, a top-down technique, has proven to be cost-effective for producing nanoparticles of various shapes and size distributions within a short time (typically a few minutes) [41]. Pulsed laser ablation can be used to synthesize Au-Ag BNPs in liquid through several preparation pathways. Initially, single-metal colloids (e.g., Ag cubes) are synthesized from ablation targets. The target is then replaced with a different metal (e.g., Au cubes), and ablation occurs under the existing colloid to produce bimetallic nanoparticles. This typically results in core–shell bimetallic nanoparticles, where the core consists of the first ablation material and the shell is composed of the second material. Additionally, Au-Ag BNPs can be synthesized by mixing two different metal colloids and ablating the mixture, resulting in alloy or core–shell bimetallic nanoparticles [42].

Au-Ag BNPs can also be produced via mechanical grinding and ball milling. For example, Murugadoss et al. mechanically mixed metal salts and reducing agents (AgNO_3_, chitosan and NaOH) in a solid state, then added a concentrated HAuCl_3_ solution to obtain Au-Ag BNPs [43].

Microwave radiation-assisted synthesis provides certain advantages for the manufacture of BNPs. Using a microwave reactor, Au-Ag BNPs were synthesized by heating a mixture of AgNO_3_ and NaAuCl_4_ in ethylene glycol and glycerol [44]. Although this physical method does not involve toxic chemicals, it is not recommended for the synthesis of Au-Ag BNPs due to its high cost, susceptibility to radiation, high temperature, low yield and high energy consumption.

#### 2.2.2. Chemical Methods

Chemical methods involve reactions with precursors and typically include co-reduction, seed-mediated growth, electrochemical displacement reactions, co-precipitation and polyol methods. Each method has distinct advantages and disadvantages, which influence the properties and morphologies of the synthesized Au-Ag BNPs. The co-reduction method simultaneously reduces Au and Ag precursors to form Au-Ag BNPs. Common precursors include HAuCl_4_ and AgNO_3_, which are dissolved in either water or an organic solvent, such as ethanol. Reducing agents, such as NaBH_4_ and trisodium citrate, provide electrons to reduce Au and Ag ions into Au and Ag nanoparticles. By adjusting the precursor concentration and the reducing agent-to-precursor ratio, nanoparticles of varying sizes can be synthesized [45].

The seed-mediated growth method involves two steps: synthesis of seed nanoparticles and the subsequent growth of a second metal on these seeds. For example, small Au NPs can be synthesized by reducing HAuCl_4_ and then used as seeds. In the second step, these seed nanoparticles are introduced into a solution containing Ag ions, such as AgNO_3_. Reducing agents facilitate the growth of Ag on the seed surface, resulting in Au-Ag BNPs with a core–shell structure [46].

Electrochemical displacement reactions utilize sacrificial templates or Ag seeds. The template is immersed in a solution containing Au ions, such as HAuCl_4_. More active Ag atoms are oxidized, releasing electrons, while Au ions are reduced and deposited onto the surface of Ag. This process leads to the gradual substitution of Ag by Au, forming Au-Ag BNPs in which Au replaces the Ag template. The size, shape and composition of the BNPs can be controlled by adjusting reaction conditions, such as the concentration of Au ions and the reaction time [47].

The polyol method uses polyol solvents, such as ethylene glycol, as both solvents and reducing agents. Au and Ag precursors are dissolved in the polyol solvent and heated to a specific temperature, initiating their reduction. The polyol solvent provides electrons to the Au and Ag ions, reducing them into nanoparticles. The reduction process can be controlled by adjusting the reaction temperature and time, resulting in nanoparticles composed of Au and Ag atoms [48].

Chemical methods offer several advantages, including the ability to synthesize a variety of nanostructures (e.g., core–shell, alloys); excellent control over size, shape, properties and surface functionality; and high-yield manufacturing. However, these methods often involve toxic chemicals, produce harmful by-products and are not environmentally friendly.

#### 2.2.3. Biological Green Synthesis Methods

Green synthesis focuses on the use of reagents that are harmless to the environment and emphasizes biological raw materials as the source of reagents for the synthesis of nanomaterials [49]. Alloys, mixtures or core–shell nanoparticles can be generated by reducing or co-reducing precursors with these biological reducing agents. Researchers have reported studies on the synthesis of BNPs using plant-based materials, including palm tree leaf extract [50], piper betle leaf extract [51], banana peel extract [52], etc.

For example, Elemike et al. reported the green synthesis of AgNPs, AuNPs and Ag-Au BNPs using *Stigmaphyllon ovatum* leaf extract in an aqueous medium [53]. UV-vis spectroscopy revealed surface plasmon resonance bands at 420 nm (AgNPs), 550 nm (AuNPs) and 542 nm (Ag-Au BNPs), with the single band in BNPs indicating nanoalloy formation. TEM analysis showed average particle sizes of 24 nm (AgNPs), 80 nm (AuNPs) and 15 nm (Ag-Au BNPs). Another study by Ganaie et al. demonstrated the eco-friendly synthesis of bimetallic Au-Ag nanoparticles (BNPs) using the invasive plant species *Antigonon leptopus* [54]. This plant’s extracts, rich in phenolic compounds, flavonoids and proteins, were utilized for reduction and stabilization of the nanoparticles. By adjusting factors like pH, temperature and reactant stoichiometry, the researchers were able to synthesize both nanoalloys and core–shell structures, such as Au-core/Ag-shell particles. These examples highlight the potential of plant-based extracts and green synthesis methods in producing nanoparticles with controlled properties, offering an eco-friendly alternative to traditional chemical approaches.

In conclusion, Au-Ag alloy nanoparticles can all be synthesized through physical, chemical and biological methods. Au-Ag core–shell nanomaterials can be synthesized through physical and chemical methods. Au-Ag aggregates are mainly synthesized through chemical methods. At present, among the three types of synthetic methods, chemical methods are more diverse, have a wider range of applications and are more commonly used for the synthesis of Au-Ag BNPs.

## 3. Application of Au-Ag SERS Substrates with Bimetallic Synergistic Effects

In the context of food safety, Au-Ag BNPs have shown potential for rapid, sensitive and non-destructive SERS detection of various contaminants and additives. SERS-based sensors using Au-Ag BNPs can detect pesticides, heavy metal ions, harmful microbes, mycotoxins and food additives (Table 1). This ability is crucial for ensuring the safety and quality of food, as it can identify and quantify potentially harmful substances before they reach consumers.

### 3.1. Detection of Harmful Microbes

Harmful microbes, particularly pathogenic bacteria, can cause food poisoning or be transmitted through food. Numerous types of foodborne pathogenic bacteria can survive and spread in various environments, posing a significant threat to human health [108].

Using Au-Ag BNP substrates and leveraging the characteristic peaks of different microorganisms, along with multivariate statistical analysis, enables the rapid and accurate detection of pathogenic microorganisms such as *Escherichia coli* and *Staphylococcus aureus* (*S. aureus*) [109]. Zhu et al. developed a multifunctional, ultrasensitive SERS film for rapid detection of foodborne pathogens in beef [61]. The substrate was constructed by self-assembling vancomycin (Van)-modified Au@Ag nanoparticles (Au@Ag NPs) onto a polydimethylsiloxane (PDMS) film, integrating the bacterial capture capability of Van with the the Raman enhancement effect of Au@AgNPs. The Au@AgNPs/Van-PDMS film exhibited a high Raman enhancement factor of 1.09 × 10^5^ and good reproducibility. It could effectively capture *Clostridium perfringens*, *Bacillus subtilis* and *S. aureus* isolated from beef, and the SERS spectra of these pathogens showed significant differences. Principal component analysis (PCA) and linear discriminant analysis (LDA) were used for classification, achieving 100% correct qualitative identification by LDA. For quantitative detection, the limit of detection (LOD) for *S. aureus* in beef was as low as 3 CFU/mL, with a high capture efficiency of 94.97% and a good linear relationship (R^2^ = 0.96). This method provides an effective means for the rapid and sensitive detection of foodborne pathogens in complex food matrices without the need for in vitro bacterial culture, showcasing great potential for practical applications in food safety monitoring. However, PDMS film may be interfered with by complex matrices.

**Figure 3:**
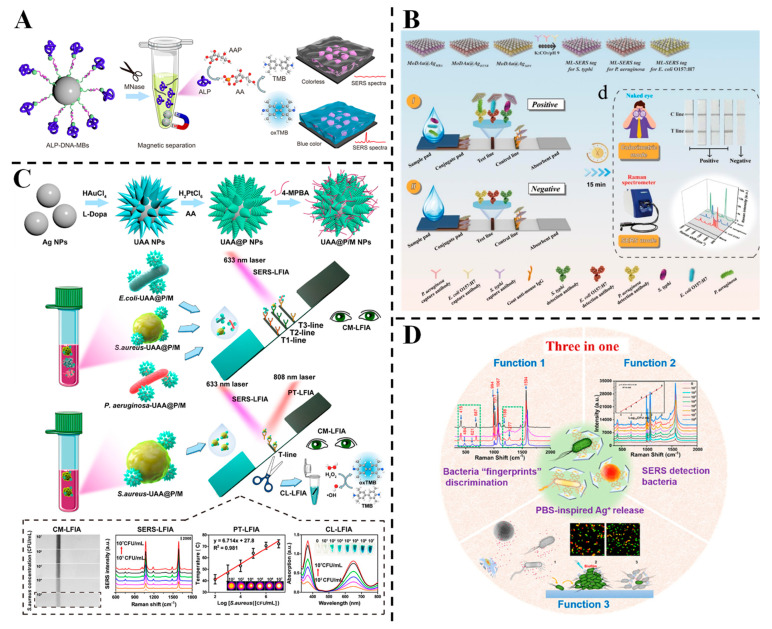
(**A**) Colorimetric/SERS sensing for micrococcal nuclease (MNase)-responsive detection [62]. (**B**) Schematic of the preparation of three kinds of immuno-MoDAu@Ag SERS tags and MoDAu@Ag-based SERS encoding-LFIA for simultaneous detection of *P. aeruginosa*, *E. coli* O157:H7 and *S. typhi* [63]. (**C**) Schematic illustration of the synthesis process for multifunctional UAA@P/M and the procedures of UAA@P/M-integrated LFIA for multimodal bacterial detection [111]. (**D**) Schematic diagram of the SERS sandwich structure made of bacteria/SERS tags/AAS-NPs, used for specific bacterial identification, sensitive detection and reliable bacterial inactivation [64].

Multimodal signal sensing methods offer complementary advantages, enhancing the reliability of results, and have garnered significant interest in the fields of nanomaterials and sensing [79,110]. Huang et al. designed a close-packed Au@AgPt nanozyme array coupled with a cascade triggering strategy for detecting *S. aureus* in serum [62] (Figure 3A). The trimetallic nanozymes catalyzed TMB oxidation to generate SERS-active oxTMB, while micrococcal nuclease-secreted bacteria triggered signal attenuation via alkaline phosphatase-mediated reduction. This “on-to-off” strategy achieved LODs of 38 CFU/mL (colorimetric) and 6 CFU/mL (SERS), with linear ranges spanning five orders of magnitude. Shen et al. proposed a 3D membrane-like (ML) tag, MoDAu@Ag, integrated with lateral flow immunoassay (LFIA) for ultrasensitive multiplex pathogen detection [63] (Figure 3B). The tag, which is constructed by loading Au and Ag core–shell nanoparticles (Au@Ag NPs) onto polyethyleneimine (PEI)-modified MoS_2_ nanosheets, enables the simultaneous detection of *P. aeruginosa*, *S. typhimurium* and *E. coli* O157:H7 using three Raman molecules. Rapid qualitative (colorimetric) and quantitative (SERS) detection of different pathogens was achieved through visual color signal evaluation and characteristic Raman signal measurement within a single detection area. The SERS-encoding LFIA achieved LODs of 30~40 cells/mL across four orders of magnitude, with stable performance in clinical, food and environmental samples. This strategy overcomes the limitations of traditional LFIA, offering a promising platform for on-site, multiplex pathogen diagnosis. Huang et al. introduced multifunctional urchin-shaped Au-Ag@Pt nanoparticles (UAA@P NPs) integrated with LFIA for multimodal bacterial detection [111] (Figure 3C). The UAA@P/M NPs, functionalized with 4-mercaptophenylboronic acid (4-MPBA), enabled ultrasensitive SERS-LFIA, PT-LFIA and CL-LFIA with LODs of 3, 27 and 18 CFU/mL, respectively—330-fold, 37-fold and 55-fold more sensitive than visual CM-LFIA. Partial least-squares discriminant analysis (PLS-DA) accurately differentiated *E. coli*, *S. aureus* and *P. aeruginosa* in spiked blood samples, with recoveries of 90.3~108.8%. These multi-mode sensing strategies have good stability, anti-interference ability and high flexibility, but they are also prone to disadvantages such as more complex material preparation and increased detection processes.

With the continuous advancement of SERS substrate fabrication technology, specific functionalization treatments of precious metal nanoparticles, combined with statistical analysis methods, enable the identification, trace detection and inactivation of pathogenic microorganisms [20]. Zhou et al. reported the development of smart triple-functional Au-Ag-stuffed nanopancakes (AAS-NPs) for simultaneous SERS-based bacterial detection, discrimination and inactivation [64] (Figure 3D). The AAS-NPs were synthesized by Ag-etching Au@Ag NPs with K_3_[Fe(CN)_6_], forming unstable Prussian blue analogues to bind bacteria via cyano groups. Using 4-MPBA as both an SERS tag and internal standard, the platform achieved highly sensitive discrimination of *E. coli*, *S. aureus* and *P. aeruginosa*, with a detection limit of 7 CFU mL^−1^. The SERS sandwich structure (bacteria/4-MPBA/AAS-NPs) provided specific “fingerprint” spectra, while PBS-stimulated AAS-NPs released (Ag^+^) to kill > 99% of 1 × 10^5^ CFU mL^−1^ bacteria within 60 min, with antibacterial activity enhanced by 64~72-fold compared to untreated nanoparticles. Although the preparation process may be more complex, intelligent SERS substrates like AAS-NPs, which have multiple functions (identification, detection, inactivation, etc.) and achieve the integration of “detection-processing”, are an important development direction for SERS substrates in the future.

### 3.2. Detection of Mycotoxins

Mycotoxins are toxic secondary metabolites produced by various fungi and are commonly found in foods such as grains, fruits and vegetables [112,113]. These toxins are particularly prone to production under favorable temperature and humidity conditions during food processing, storage and transportation. Recent advancements in nanotechnology have led to the development of various Au-Ag BNPs substrates, which significantly enhance SERS signals, improve detection limits and make this technology more applicable to real-world samples [114]. Consequently, SERS has emerged as an active research area for the detection of mycotoxins, including aflatoxin, deoxynivalenol (DON), zearalenone (ZEN) and patulin (PAT). Efforts are ongoing to develop novel nanomaterials and methodologies for more sensitive and rapid analysis [115,116].

**Figure 4:**
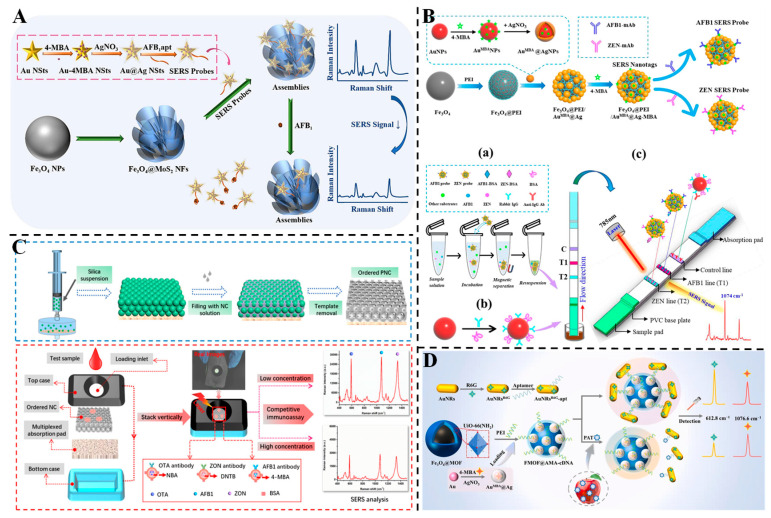
(**A**) Schematic illustration of the developed 3D SERS aptasensor based on Au-4MBA@Ag NSts-AFB_1_apt- Fe_3_O_4_@MoS_2_ NFs assemblies for AFB_1_ detection [69]. (**B**) Schematic diagrams of the preparation of two SERS probes and the SERS-LFIA test strip sensing process for simultaneous detection of two mycotoxins [72]. (**C**) SERS VFA biosensor based on an ordered PNC membrane and SERS nanotags for multiplex mycotoxin detection [73]. (**D**) Schematic diagram of the preparation process and sensing principle of magnetic MOFs-based ratiometric SERS aptasensor [74].

Aflatoxins are a common group of mycotoxins, comprising at least 18 known types. Among them, aflatoxin B_1_ (AFB_1_) is the most toxic and carcinogenic, making it a major focus of regulatory concern in agricultural products [117]. To enable ultrasensitive detection of AFB_1_, Chen et al. developed a three-dimensional (3D) plasmonic SERS aptasensor by integrating Au@Ag bimetallic nanostars (Au@Ag BNSs) with Fe_3_O_4_@MoS_2_ magnetic nanoflowers [69]. As illustrated in Figure 4A, the Au@Ag BNSs, functionalized with 4-mercaptobenzoic acid (4-MBA) and AFB_1_-specific aptamers, served as SERS probes, while the Fe_3_O_4_@MoS_2_ component enabled magnetic separation and signal enhancement via synergistic plasmonic and chemical effects. The sensor demonstrated a low LOD of 58.9 pg/mL and a linear detection range from 0.1 to 100 ng/mL. In spiked peanut samples, the recovery rates ranged from 95.53% to 98.73%, with RSDs below 5.2%, indicating high specificity and minimal matrix interference. The 3D architecture facilitated efficient signal enrichment and rapid magnetic separation, offering a promising approach for on-site AFB_1_ detection in food safety applications. Similarly, Tan et al. designed a dual-mode aptasensor combining colorimetric and label-free SERS detection for ultrasensitive quantification of AFB_1_ [70]. The sensor employed self-assembled Ag@Au IP_6_ bifunctional nanozymes, whose core–shell structure provided both peroxidase-like activity for TMB–H_2_O_2_ color reactions and plasmonic enhancement for SERS signal amplification. The aptasensor leveraged a hybridization chain reaction to amplify signal molecules, achieving an LOD of 0.58 pg/L and a linear range from 2 to 200 pg/L. In spiked red wine samples, recoveries ranged from 97.12% to 101.28% with RSDs within 5%, demonstrating resistance to matrix interference. The dual-mode strategy combined a visual colorimetric readout with quantitative SERS analysis, offering a robust platform for on-site and precise AFB_1_ monitoring in complex food matrices.

DON contamination not only reduces grain quality but also induces symptoms such as vomiting, anorexia and immunotoxicity and disrupts growth and reproduction [118]. Zhao et al. developed a core–shell–satellite nanoassembly consisting of an AuNR@Ag core, an ultrathin SiO_2_ layer and AuNP satellites, which served as an SERS immunoprobe for ultrasensitive detection of DON [71]. The anisotropic AuNR@Ag core facilitated strong localized electromagnetic coupling, while the SiO_2_ layer protected the Raman reporter (4-MBA) and enhanced colloidal stability. The AuNP satellites increased surface coverage and hotspot formation, thereby improving signal amplification. The SERS-LFIA exhibited an LOD of 0.053 fg/mL and a wide linear range from 0.1 fg/mL to 1 μg/mL. In spiked corn and wheat samples, recoveries ranged from 95.90% to 105.83%, with RSDs < 7.67%, demonstrating excellent accuracy and resistance to matrix interference. This strategy integrates LFIA and plasmonic enhancement, offering a robust platform for rapid, quantitative DON detection in complex food matrices, with potential applications for trace mycotoxin analysis in food safety monitoring.

ZEN has been identified as an endocrine disruptor that can impair the development of internal organs, lead to reproductive disorders and cause digestive dysfunction in animals, posing significant economic risks [119]. Humans are also at risk of ZEN exposure through the consumption of contaminated food. Therefore, the effective detection of ZEN is crucial for safeguarding human health [120]. Furthermore, the coexistence of multiple mycotoxins in food is common, and the potential synergistic effects and enhanced toxicity from their accumulation present serious threats to both human health and the economy [121]. Yin et al. developed a magnetic nanocomposite-based SERS-LFIA sensor for the simultaneous detection of AFB1 and ZEN in corn samples [72] (Figure 4B). The core–interlayer–satellite magnetic nanocomposites (Fe_3_O_4_@PEI/Au^MBA^@Ag^MBA^) were employed as dual-functional SERS tags, integrating magnetic enrichment and Raman signal amplification. Under optimal conditions, the detection ranges of AFB1 and ZEN in corn samples were 0.1~10 μg/kg and 4~400 μg/kg, with LODs of 0.095 μg/kg and 1.896 μg/kg, respectively. The recoveries of spiked corn samples ranged from 91.28% to 109.52% for AFB1 and 94.71% to 108.15% for ZEN, with RSDs < 10%. The method showed good agreement with HPLC results and enabled rapid detection within 20 min, demonstrating its potential for on-site, simultaneous monitoring of mycotoxins with high sensitivity, accuracy and practicality. SERS technology offers advantages such as a sharp peak shape, high peak intensity and high sensitivity. When combined with various Raman tag molecules for SERS encoding, it facilitates multiplexed detection at a single site. As shown in Figure 4C, Chen et al. developed an SERS vertical flow assay (VFA) using photonic nitrocellulose (PNC) with an ordered inverse opal structure as the sensing substrate for simultaneous detection of ochratoxin A (OTA), AFB_1_ and ZEN [73]. Three SERS nanotags (Au^NBA^@Ag, Au^4-MBA^@Ag and Au^DNTB^@Ag) were encoded with distinct Raman reporters for each mycotoxin, enabling multiplexed detection. The slow-photon effect of the PNC substrate enhanced light–matter interaction, boosting SERS signal intensity. The assay achieved LODs of 8.2 fg/mL for OTA, 13.7 fg/mL for AFB1 and 47.6 fg/mL for ZEN, all below European Commission tolerable limits. Spiked recoveries in cereal samples ranged from 89.6% to 107.1%, consistent with ELISA results. The ordered porous structure improved reagent mixing and immune reaction kinetics, demonstrating the potential of the PNC-based VFA for high-throughput, on-site screening of multiple mycotoxins in food safety applications.

PAT causes damage to both the respiratory and urinary systems, resulting in nerve palsy, pulmonary edema and renal failure. It is also teratogenic, carcinogenic and highly toxic to human health [122,123]. Zhou et al. developed a magnetic metal–organic framework (MOF)-based ratiometric SERS aptasensor for the sensitive detection of PAT in apples [74] (Figure 4D). The sensor utilized Fe_3_O_4_@UiO-66-NH_2_ loaded with 4-MBA-labeled Au@Ag NPs as the SERS substrate, and AuNRs modified with rhodamine 6G (R6G) and aptamers as capture probes. The magnetic MOF facilitated efficient separation and enrichment of target analytes, while the dual-layer Raman reporter system ensured stable internal calibration. The SERS intensity ratio exhibited a negative correlation, with PAT concentrations ranging from 0.01 to 100 ng/mL, with an LOD of 0.0465 ng/mL. In spiked apple samples, recoveries ranged from 95.90% to 105.83%, demonstrating high resistance to interference and accuracy. The integration of magnetic separation and ratiometric sensing mitigates matrix effects, highlighting the aptasensor’s potential for practical mycotoxin detection in real food samples.

### 3.3. Detection of Pesticides

The use of pesticides has significantly enhanced agricultural productivity [124]. However, the widespread application of pesticides in agriculture has also led to serious pesticide residues, which pose inevitable risks to both the ecosystem and human health [125]. Consequently, the development of simple, rapid, sensitive and reliable detection methods for the swift monitoring of pesticide residues in food has become an urgent challenge. SERS technology is widely used in pesticide residue analysis due to its advantages, including non-destructive data acquisition, high sensitivity and fast detection speed [126,127].

Au-Ag alloys nanoparticles exhibit a synergistic effect between Au and Ag and a good SERS enhancement effect. As a result, many pesticide molecules with high SERS sensitivity are typically detected using label-free methods. Cho et al. synthesized silica nanoparticles surface-decorated with Au-Ag alloy nanoparticles (SiO_2_@AuAg) as stable SERS substrates [80]. Seed-mediated growth was used to control the Au:Ag ratio, optimizing hotspots for enhanced Raman signals. The substrates demonstrated high sensitivity with LODs of 6.95 × 10^−7^ M (crystal violet), 5.56 × 10^−7^ M (thiram) and 7.14 × 10^−6^ M (carbaryl), along with excellent reproducibility (RSD ≈ 8.4% after 3 days). The robust structure combined silica stability with Au-Ag plasmonic effects, enabling reliable detection of trace contaminants in environmental and food samples.

In addition, spectral data often contains noise from the sample substrate. Chemometric algorithms are valuable tools for extracting meaningful spectral information. Deep learning has advanced significantly in the field of chemometrics and has been successfully applied to the self-learning and modeling of spectroscopic data [128]. Li et al. designed Au-Ag octahedral hollow cages (Au-Ag OHCs) using Cu_2_O templates and coupled them with a convolutional neural network (CNN) for quantifying thiram and pymetrozine in tea [34]. The hollow structure and rough edges of Au-Ag OHCs enhanced electromagnetic fields, while the CNN algorithm improved spectral analysis, achieving LODs of 0.286 μg/kg (thiram) and 29 μg/kg (pymetrozine). The CNN model outperformed partial least squares and extreme learning machine, with correlation values reaching 0.995, demonstrating robust anti-interference capabilities and accuracy comparable to HPLC. The machine learning (especially CNN) in this method significantly improves the ability of complex spectral analysis, but the problems of model lightweighting and on-site deployment need to be solved.

**Figure 5:**
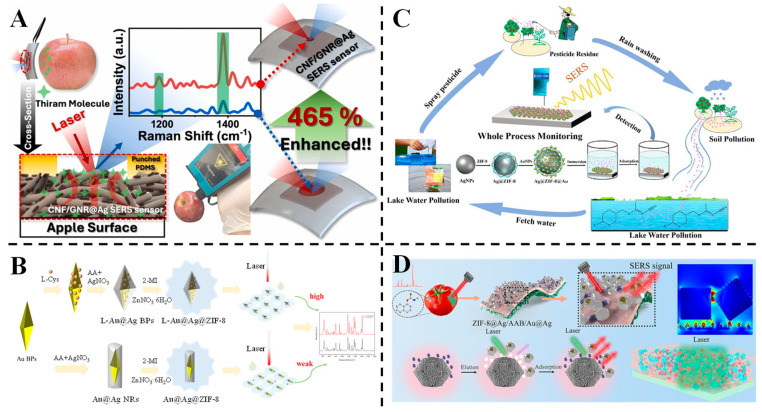
(**A**) Schematic diagram of detection of pesticides on apple surfaces using CNF/GNR@Ag SERS substrate [81]. (**B**) Schematic overview of the synthesis of AAZ and L-AAZ and the working principle of the SERS sensor for quinalphos detection [82]. (**C**) Illustration of the fabrication of Ag@ZIF-8@Au and its application in SERS detection of acetamiprid [83]. (**D**) Schematic diagram of paper-based SERS sensor quantification of carbaryl [84].

Au-Ag core–shell nanomaterials also have significant Raman enhancement effects and are widely used in pesticide residue detection. For example, Park et al. developed a flexible cellulose nanofiber (CNF)/AuNRs@Ag (Au@Ag NRs) SERS sensor for on-site pesticide detection, utilizing localized evaporation enrichment via hole-punched PDMS [81] (Figure 5A). The hydrophilic CNF matrix and Ag-coated AuNRs created dense hotspots, achieving an ultra-low LOD of 10^−11^ M for thiram on apple and chili pepper surfaces. The sensor demonstrated a 465% signal enhancement via controlled evaporation, overcoming coffee-ring effects and improving analyte concentration in the detection area. Portable Raman spectroscopy enabled real-time analysis, highlighting its adaptability for non-planar surfaces and complex food matrices. Raveendran and Docoslis developed a two-step method to fabricate Ag-Au core–shell nanostructures on microelectrodes for SERS-based toxicant detection [129]. Ag nanostructures were first grown via electrochemical deposition on microelectrodes functionalized with 11-mercaptoundecanoic acid, followed by a galvanic reaction with HAuCl_4_ to form the Au shell. The Ag-Au nanostructures exhibited a maximum SERS enhancement factor of 6.51 × 10^5^ and showed superior chemical stability, retaining 93 ± 7.3% of the signal after 24 h in PBS compared to 48 ± 5.0% for Ag nanostructures. Using multivariate analysis with classifiers, they successfully detected and quantified four toxicants (thiram, thiabendazole, malachite green and biphenyl-4-thiol) at 1 ppm with 100% accuracy. Additionally, flexible Ag-Au/PDMS SERS substrates were fabricated, enabling direct detection of thiram on apple peels without sample pretreatment, demonstrating their potential for on-site food and water safety monitoring.

MOFs, with their large surface area, are highly effective in adsorbing target substances [130]. They are increasingly being integrated with precious metal nanomaterials to create composite structures for novel SERS substrates. Yang et al. fabricated a chiral spiny L-Au@Ag@ZIF-8 (L-AAZ) three-layer core–shell SERS substrate for quinalphos detection in tangerines [82] (Figure 5B). The chiral L-Au@Ag bipyramids provided abundant hotspots, while ZIF-8 facilitated selective adsorption and molecular sieving, thereby reducing matrix interference. This substrate achieved a high enhancement factor (3.15 × 10^5^) and a low LOD of 6.56 × 10^−10^ M for malachite green, with stable performance over 55 days. Density functional theory (DFT) simulations and X-ray photoelectron spectroscopy (XPS) analysis revealed that hydrogen bonding and π-π interactions were primarily responsible for the adsorption process. Real-sample testing demonstrated an LOD of 10 ng/mL in tangerine juice, confirming its potential for sensitive food safety monitoring. Zhang et al. fabricated Ag@ZIF-8@Au nanoparticles as a robust SERS platform for acetamiprid detection [83] (Figure 5C). The core–shell structure integrated ZIF-8’s molecular enrichment capabilities with Ag/Au plasmonic hotspots, achieving an LOD of 9.027 × 10^−10^ M and a high enhancement factor of 4.3 × 10^7^. The platform exhibited excellent reproducibility (RSD ≤ 7.198%) and stability (RSD = 3.127% over 6 weeks), effectively detecting acetamiprid in complex matrices such as lake water, tea leaves and oranges. The synergistic electromagnetic and chemical enhancements, combined with ZIF-8’s adsorption capability, enabled reliable trace detection, underscoring its potential for on-site applications. Pan et al. constructed a ZIF-8@Ag/AAB/Au@Ag composite SERS paper sensor for carbaryl quantification, leveraging a 3D platform with cellulose paper as the substrate [84] (Figure 5D). ZIF-8 facilitated molecular adsorption, Ag layers enhanced plasmonic hotspots and Au@Ag NPs boosted electromagnetic coupling. The sensor demonstrated a linear range of 0.01~20 µg/mL and a low LOD of 5.72 × 10^−3^ µg/mL, with high reproducibility (RSD = 4.49%) and stability over 2 months. Finite-difference time-domain (FDTD) simulations confirmed enhanced electromagnetic fields at the Ag/Au@Ag NP interface, validating the full-dimensional divergence enhancement strategy for rapid on-site detection in fruits and vegetables. FDTD simulation provides a new idea for optimizing the structure of SERS nanomaterials or confirming the “hotspots” distribution of nanomaterials.

### 3.4. Detection of Antibiotics

Antibiotics are extensively used in industries such as aquaculture, livestock and poultry production, and agriculture to enhance economic productivity. Consequently, antibiotic residues are frequently detected in various food products, including meat, fish, milk, eggs and fruits. These residues accumulate in the human body through the food chain, potentially causing damage to organs and leading to conditions such as anemia and cardiovascular diseases [131]. Therefore, establishing an effective method for monitoring and analyzing veterinary drug residues in aquatic products is crucial for ensuring food safety.

**Figure 6:**
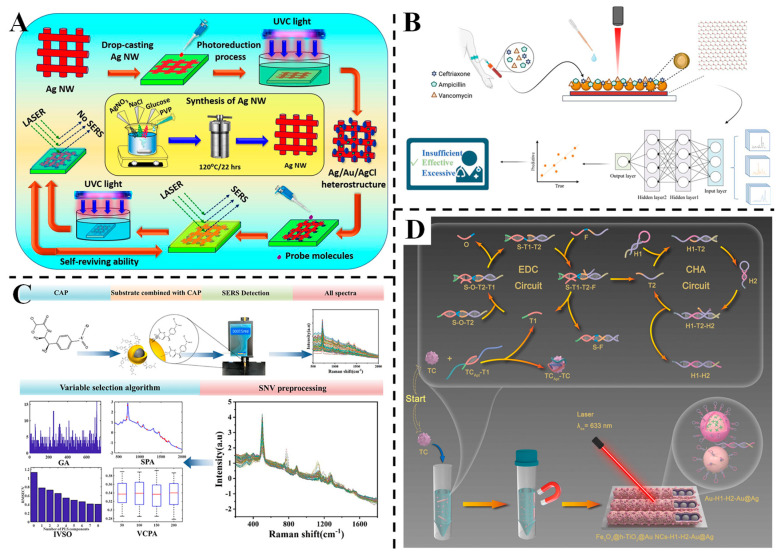
(**A**) Schematic representation of the synthesis procedure, the SERS sensing and the self-reviving ability for the SERS substrate based on the Ag/Au/AgCl heterostructure [89]. (**B**) Schematic diagram of detection and analysis of three antibiotics [91]. (**C**) Schematic diagram of chloramphenicol detection [92]. (**D**) Schematic diagram of TC detection based on aptamer recognition and cascade DNA network amplification Raman aptasensor [93].

Au-Ag nanoaggregates create nanoscale gaps that facilitate the generation of strong Raman hotspots, thereby enhancing detection sensitivity. Barveen et al. developed an Ag/Au/AgCl heterostructure via hydrothermal and photoreduction processes, serving as a reusable SERS substrate for ultrasensitive detection of analgesics and antibiotics [89] (Figure 6A). The nanoscale interparticle gaps in the heterostructure generated abundant hotspots, enhancing Raman signals through synergistic electromagnetic and chemical effects. The substrate exhibited wide linear ranges for paracetamol (10^−1^~10^−10^ M) and furazolidone (10^−1^~10^−9^ M), with detection limits as low as 2.8 × 10^−12^ M and 1.9 × 10^−11^ M, respectively. It enabled separate and multiplex detection in human urine samples with satisfactory recoveries (80~105%). The AgCl component facilitated photodegradation of adsorbed molecules, allowing the substrate to maintain ~84.2% SERS activity after five recycling tests, highlighting its potential for accurate and sustainable trace detection in complex biological matrices. Jiao et al. constructed aligned TiO_2_ nanorod arrays (NRAs) decorated with closely interconnected Au/Ag nanoparticles, creating a near-infrared (NIR) SERS active sensor for antibiotic detection in water [90]. The heterostructure exhibited strong absorption in the 400~1300 nm region, enabling ultrasensitive 785 nm laser-excited detection of ciprofloxacin (LOD = 10^−9^ M) and chloramphenicol (LOD = 10^−8^ M) in real-world water samples. The unique architecture provided abundant hotspots and efficient charge transfer, enhancing both electromagnetic and chemical contributions to SERS. Leveraging the photocatalytic degradation capability of TiO_2_, the sensor retained ~84.2% of its SERS activity after five cycles, demonstrating excellent reusability. This work presents a multifunctional NIR-SERS platform with high sensitivity and sustainability for monitoring antibiotic residues in environmental and biological matrices.

Cao et al. designed a highly sensitive SERS biosensor using WS_2_/Au@Ag nanocomposites for detecting ceftriaxone, ampicillin and vancomycin in serum, integrated with a 2D-CNN deep learning model for concentration prediction [91] (Figure 6B). The hybrid substrate, combining two-dimensional WS_2_ and noble metal core–shell nanostructures, achieved an LOD of 10^−14^ M for R6G and exhibited linear responses for the three antibiotics across 0.5~1000 μg/mL. The 2D-CNN model, which converts SERS spectra into 2D images via the short-time Fourier transform, demonstrated excellent regression performance with R^2^ values of 0.9993 and 0.9997 for ceftriaxone and ampicillin in mixed serum solutions, surpassing traditional machine learning methods. Yang et al. designed a rapid method for chloramphenicol (CAP) residue detection in tilapia using Ag@Au NPs as SERS nanosensors coupled with chemometric algorithms [92] (Figure 6C). The Ag@Au NPs exhibited a high enhancement factor (2.67 × 10^6^), and the variable combination population analysis-partial least-squares (VCPA-PLS) model with standard normal variate pretreatment achieved an LOD of 1 × 10^−5^ μg/mL. The method showed excellent predictive performance, with recoveries ranging from 98 to 104% in real samples, demonstrating its reliability and applicability for sensitive and specific CAP determination in complex food matrices.

Lv et al. constructed a cascade amplification SERS aptasensor for tetracycline (TC) detection using Fe_3_O_4_@h-TiO_2_/Au nanochains and Au@Ag NPs integrated with enzyme-free DNA circuits [93] (Figure 6D). The dual amplification strategy enabled a low LOD of 15.91 pg/mL and a linear range from 0.01 to 100 ng/mL. The magnetic Fe_3_O_4_ facilitated easy separation, while the Au@Ag NPs provided strong SERS enhancement. The sensor exhibited excellent specificity and storage stability, with recoveries in real samples (fish, milk and lake water) ranging from 93.2 to 105.3%, showcasing its utility for sensitive and efficient TC analysis in food and environmental safety.

### 3.5. Detection of Heavy Metals

Heavy metal ions accumulate in organisms through the food chain within the ecological cycle, posing significant harm to animals, plants and humans [132]. In recent decades, the threat posed by food contaminated with heavy metals has raised substantial concern among the public [133]. Once absorbed, these ions can bind to proteins, leading to their inactivation and compromising the health and safety of organisms. Consequently, the rapid, cost-effective and accurate detection of heavy metal ions is crucial for human health monitoring and environmental protection [134].

Xu et al. developed a highly sensitive SERS sensor for ultratrace detection of Cr (VI), utilizing methimazole-functionalized Au@Ag nano-sea urchins (Au@Ag NSUs) integrated with paper tips [94]. The Au@Ag NSUs combined the stability of Au cores with the strong SERS enhancement provided by the Ag shells. The paper tip facilitated gravity-driven analyte enrichment at the tip, significantly amplifying the signal. The redox reaction between methimazole and Cr (VI) modulated the SERS signal intensity, enabling an LOD as low as 0.956 ng/L. The sensor demonstrated excellent reproducibility (RSD ≤ 7.35%) and stability, retaining 92% of its initial signal intensity after 30 days. Analysis of real water samples, including lake and tap water, yielded recoveries between 98.17% and 105.73%, confirming its reliability for environmental monitoring. This work underscores the potential of paper-based SERS platforms for on-site, cost-effective heavy metal analysis.

**Figure 7:**
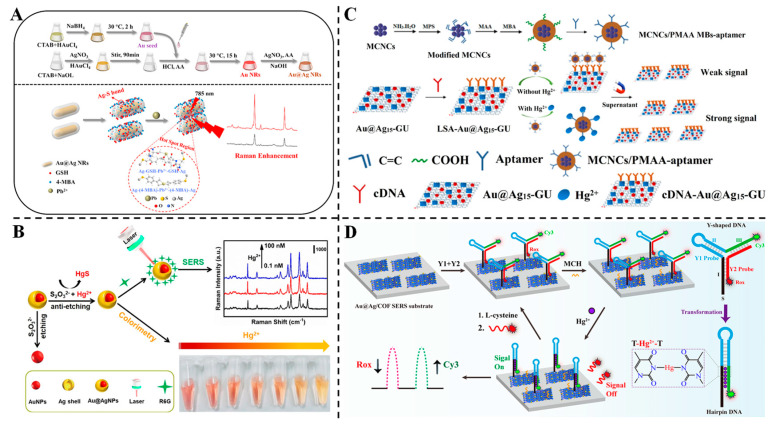
(**A**) Process of preparing Au@Ag NRs and schematic illustration of the detection of Pb^2+^ based on the aggregates of the Au@Ag NR probe [95]. (**B**) Principle diagram of the colorimetric and SERS dual-mode probe for determination of Hg^2+^ based on controllable etching unmodified Au@Ag NPs [96]. (**C**) Dual-channel biosensor based on Au@Ag15-GU nanohybrids for detection of Hg^2+^ [97]. (**D**) Schematic illustration of the dual-signaling SERS ratiometric platform for Hg^2+^ detection [98].

Liu et al. designed an SERS sensor for Pb^2+^ detection using glutathione and 4-MBA-functionalized Au@Ag core–shell nanorods (Au@Ag NRs) [95] (Figure 7A). The free carboxyl groups of glutathione and 4-MBA chelated Pb^2+^, inducing nanoparticle aggregation and enhancing “hotspots” for SERS signal amplification. Under optimized conditions, the sensor showed a linear response from 0.5 to 1000 μg/L, with an LOD of 0.021 μg/L and a high enhancement factor (1.0328 × 10^7^). Real samples, including tea powder and glutinous rice flour, exhibited recoveries of 81.31~101.12% and low RSDs (≤9.82%), verifying its accuracy for food safety monitoring. The strategy combines specific chelation with plasmonic enhancement, enabling rapid and sensitive Pb^2+^ analysis in complex matrices.

Wang et al. proposed a dual-mode strategy for Hg^2^^+^ detection using Au@AgNPs whose Ag shells undergo controllable etching by thiosulfate ions [96] (Figure 7B). In the presence of Hg^2^^+^, the formation of insoluble HgS suppresses shell etching, restoring the orange colloidal color and enhancing the SERS signal of R6G. The colorimetric method achieved a detection limit of 2 μM (naked eye) and 0.2 μM (UV–vis), while the SERS method offered a far lower limit of 0.1 nM with a linear range of 0.1 nM~1 μM. This dual-mode approach integrates visual screening and ultrasensitive quantification, showcasing versatility for field-based and laboratory applications. Li et al. fabricated a dual-channel biosensor leveraging Au@Ag/graphene-upconversion (Au@Ag-GU) nanohybrids as multifunctional signal indicators for simultaneous SERS and fluorescence detection of Hg^2+^ [97] (Figure 7C). Magnetite–polymethacrylic acid magnetic beads (MCNCs/PMAA MBs) conjugated with aptamers captured Hg^2+^, triggering the release of cDNA-Au@Ag-GU into supernatants. The Au@Ag-GU hybrid enhanced both signals: SERS achieved a detection limit of 0.33 ppb, while fluorescence offered 1 ppb, with linear ranges of 0.1~800 nM and 0.001~100 nM, respectively. Spiked tap water and milk samples showed recoveries of 96.0~109.8% with low RSDs (≤4.76%), demonstrating its applicability for food and environmental analysis. The dual-mode design integrates the advantages of high sensitivity and a broad linear range, addressing diverse detection needs. Li et al. introduced a ratiometric SERS sensor based on Au@Ag NPs embedded in covalent organic frameworks (COFs) combined with Y-shaped DNA labeled with two Raman reporters (Cy3 and Rox) [98] (Figure 7D). The Au@Ag/COF substrate provided uniform electromagnetic fields, while the Y-shaped DNA design brought reporters closer to the surface, enhancing signal coupling. In the presence of Hg^2^^+^, the formation of T-Hg^2+^-T complexes increased Cy3 signals (“signal-on”) and decreased Rox signals (“signal-off”), resulting in an LOD of 5.0 × 10^−16^ M, among the lowest reported for Hg^2+^. Real samples (river water, tap water and milk) exhibited recoveries of 97.9~104.0% and excellent reproducibility (RSD ≤ 8.0%). This strategy integrates structural design, COF stability and dual-signal correction, offering a robust platform for ultrasensitive heavy metal detection in complex matrices.

### 3.6. Detection of Other Contaminants

Other food contaminants include food additives, nanoplastics, bacterial toxins, etc. Due to their toxicity, carcinogenicity or mutagenicity, they can cause acute and chronic health problems. Because it can achieve real-time non-destructive testing and has the advantages of high sensitivity and accuracy, SERS technology is used in the detection of these substances. Based on Au-Ag BNPs, the SERS can be functionalized and modified to achieve highly sensitive trace detection of harmful substances, further ensuring food safety.

**Figure 8:**
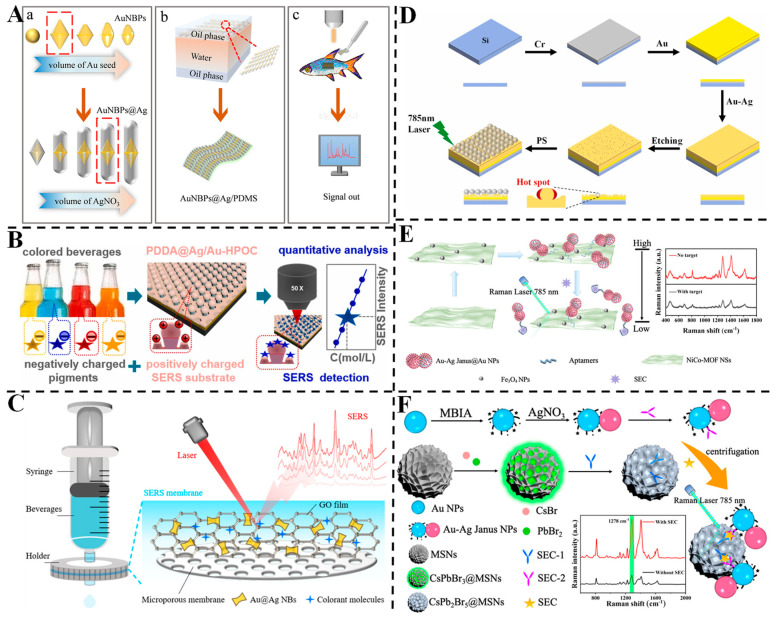
(**A**) Illustration of the preparation of Au NBPs@Ag/PDMS as SERS substrate for detecting contaminants in river bass [101]. (**B**) The detection process for weakly adsorbed dye molecules based on PDDA@Ag/Au-HPOC substrate [102]. (**C**) Schematic diagram of the enrichment procedure of colorants in beverages and SERS detection combined with GO/Au@Ag NBs membrane [103]. (**D**) Synthetic scheme of preparation procedure of the Ag@Au film SERS substrate [104]. (**E**) Schematic illustration of the SERS-active Au-Ag Janus@Au NP-engineered SERS aptasensor for the detection of SEC [105]. (**F**) Schematic illustration of plasmonic Au-Ag Janus NP/perovskite composite-engineered SERS immunoassay for SEC detection [106].

Niu et al. designed Au nanobipyramid@Ag (Au NBPs@Ag) nanorods with tunable aspect ratios via wet chemical synthesis, optimizing their plasmonic properties for enhanced SERS performance [101] (Figure 8A). By controlling the amount of Ag precursor, nanorods with an optimal aspect ratio of 4.43 demonstrated strong electromagnetic coupling, enabling highly sensitive detection of malachite green. The flexible PDMS-supported substrate, fabricated via interfacial self-assembly, achieved an LOD of 0.1 nM and a wide linear range (0.1 nM to 10 μM). When applied to river bass samples, the substrate exhibited reliable recoveries (89.8~121%) and low RSDs (<15%), highlighting its potential for environmental and food safety monitoring of trace contaminants. Zhang et al. engineered a poly(diallyldimethylammonium chloride)/Ag/Au hybrid plasmonic optical cavity (PDDA@Ag/Au-HPOC) substrate via UV holographic lithography, utilizing electrostatic interactions between positively charged PDDA and negatively charged dyes for enhanced adsorption [102] (Figure 8B). This strategy enabled direct SERS detection of dyes like amaranth and Allura red in unopened beverages without pretreatment, achieving LODs of 0.3022 and 0.2482 mg/L, respectively. The substrate exhibited excellent selectivity (no interference from neutral/positively charged molecules) and stability (signal retention over 9 days, RSD < 12.7%). A linear response (R^2^ > 0.96) and high recovery rates (95~110%) in real drinks validate its applicability for on-site food additive monitoring. Kong et al. developed a graphene oxide (GO)/Au@Ag nanobone (NB) membrane, combining the enrichment capability of GO with the plasmonic enhancement of Au@Ag for simultaneous extraction and SERS detection of colorants [103] (Figure 8C). The microporous membrane provided a large surface area for dense “hotspots” formation, achieving an LOD of 1.12 × 10^−9^ M for R6G and detecting six colorants (including carmine and brilliant blue) at concentrations below regulatory limits. A support vector machine (SVM) model enabled accurate colorant identification (94.8% accuracy), even for those with overlapping Raman peaks. Successful detection in commercial beverages (e.g., energy drinks and bayberry wine) demonstrates its potential for rapid, on-site food safety screening.

Liu et al. presented an easily fabricated Ag core embedded Au film (Ag@Au Film) for direct SERS detection of polystyrene (PS) nanoplastics [104] (Figure 8D). By optimizing the Ag-Au gap via nitric acid etching, the substrate achieved LODs of 25~50 μg/mL for PS particles (50~310 nm) in deionized and river water, with linear ranges up to 6.25 mg/mL (R^2^ > 0.97). FDTD simulations revealed enhanced electromagnetic fields at the Ag-Au interface, driving signal amplification. The substrate showed good reproducibility (RSD < 8.1%) and detected multiple plastics (PE, PET and PP), offering a simple, sensitive method for environmental microplastic analysis without complex pretreatment.

Xu et al. developed an SERS immunoassay for the sensitive detection of *Staphylococcus aureus* enterotoxin C (SEC) using Au-Ag Janus nanoparticle (NP)/perovskite composites [106] (Figure 8E). The plasmonic Au-Ag Janus NPs, functionalized with 2-mercaptobenzoimidazole-5-carboxylic acid (MBIA) ligands, exhibited inherent SERS activity. CsPbBr_3_@mesoporous silica nanomaterials (MSNs) were synthesized and transformed into CsPb_2_Br_5_@MSNs in the aqueous phase. Paired SEC antibody–antigen interactions drove the formation of Au-Ag Janus NP–CsPb_2_Br_5_@MSN composites, which showed amplified SERS activity due to electromagnetic field enhancement and electron transfer mechanisms. A linear correlation was established between the SERS signals of the composites and the SEC concentration, achieving an LOD of 0.83 pg/mL. This additive-free SERS immunoassay is simple, sensitive and reproducible, demonstrating its potential for food safety monitoring by harnessing the synergistic effects of plasmonic NPs and perovskite materials. Xu et al. reported the tunable preparation of SERS-active Au-Ag Janus@Au NPs for label-free detection of SEC [105] (Figure 8F). By adjusting the pH of the reaction system, solid and hollow Au-Ag Janus@Au NPs were synthesized, with solid structures exhibiting enhanced SERS activity due to strong plasmonic coupling between the Au dots and Au-Ag Janus NPs. The solid Au-Ag Janus@Au NPs showed a 2.27-fold higher SERS intensity than the Au-Ag Janus NPs and a 17.46-fold higher intensity than their hollow counterparts. These NPs were employed as label-free probes in an SERS aptasensor, where aptamer-modified Au-Ag Janus@Au NPs were assembled on NiCo-MOF/Fe_3_O_4_ nanosheets. The aptasensor demonstrated a low LOD of 0.55 pg/mL and excellent stability, with recovery rates in milk samples ranging from 94.4% to 98.1%. This strategy offers a sensitive and reliable method for SEC detection in complex food matrices, showcasing the advantages of tunable nanostructures for improved SERS performance.

## 4. Challenges and Outlook

SERS technology offers several advantages, including rapid detection, high sensitivity, non-destructiveness, minimal sample preparation, immunity to water interference and reduced photobleaching. Au-Ag BNPs are ideal materials for SERS substrates due to the stability of Au and the high enhancement activity of Ag. As a result, this technology has gained significant attention from researchers in the field of food contaminant detection. Its practical application in complex food matrices inevitably faces some challenges and opportunities.

### 4.1. Challenges

SERS technology using Au-Ag BNPs faces notable challenges in practical food contaminant detection. First, synthesizing Au-Ag BNPs with a uniform morphology and high stability remains problematic. Core–shell nanoparticles often exhibit irregular structures like nanorods or nanostars when prepared via seed growth methods, relying on surfactants such as cetyltrimethyl ammonium bromide (CTAB) that introduce background interference and compromise batch-to-batch repeatability. Although the Ag shell on the outside can achieve a better Raman enhancement effect than the Au shell, it also has disadvantages such as being prone to oxidation, resulting in poor long-term stability. Therefore, a protective layer can be added outside the Ag shell, such as silica, a surfactant, a polymer, etc. [135,136]. Additionally, controlling the Ag shell thickness is equally challenging. If the Ag shell is too thin (<3 nm), the localized surface plasmon resonance effect will be inadequate; if it is too thick (>10 nm), the core “hotspots” will be shielded [137]. Second, complex food matrices pose interference, as large molecules like proteins and polysaccharides adsorb onto nanoparticle surfaces, hindering target binding and decreasing Raman signals, especially in label-free applications [138]. While sample pretreatment could mitigate this issue, it increases time consumption, complicates procedures and may not meet the requirements for on-site testing. Lastly, standardization and commercialization hurdles persist, including a lack of unified substrate preparation and testing protocols across laboratories (reducing data comparability) and the high costs of Raman instrumentation and specialized synthesis of Au-Ag BNPs, restricting accessibility for small enterprises and field use.

### 4.2. Outlook

In recent years, SERS technology based on Au-Ag BNPs has also ushered in new development opportunities. First, precise control over shell structures is enabling the development of smart substrates. For example, a core–shell–satellite structure with “hotspot networks” can be formed by depositing secondary Ag NPs onto the surface of Au@Ag NPs [77]. MOFs (such as ZIF-8) or graphene coatings can be introduced to increase adsorption sites and form porous/hollow structures [139]. Magnetic composite nano-substrates can be prepared, and SERS can be combined with magnetic separation to achieve rapid enrichment and detection of target substances. The photothermal effect of Au@Ag NPs can inactivate microorganisms, facilitating integrated “detection–disinfection” processes [140]. Second, surface modification with aptamers, antibodies or molecular imprinting enhances anti-interference performance and selectivity. In label-free detection, Raman-active molecules within the Raman-silent spectral window (1800~2800 cm^−1^) are employed to enhance resistance to optical interference [141]. In addition, the geometric structure of Au-Ag BNPs significantly influences LSPR and electromagnetic field distribution. Generally, an increase in nanoparticle size leads to a redshift in the LSPR peak and an exponential enhancement of the electromagnetic field intensity. Sharp structural features can markedly amplify the local field strength due to the tip effects. Moreover, a smaller interparticle distance results in a stronger electric field, also exhibiting exponential enhancement. The dielectric properties and material composition of the nanoparticles further affect the electromagnetic field intensity. The FDTD simulation results provide valuable guidance for the rational design of SERS substrates and can verify the spatial distribution or variation in electromagnetic fields in practical applications. Furthermore, detection methodologies are advancing through the integration of portable platforms (e.g., paper-based or flexible substrates, and microfluidic chips) for on-site analysis, as well as microarrays for high-throughput screening. Finally, the incorporation of artificial intelligence (AI) algorithms facilitates automated spectral analysis and real-time decision-making, thereby promoting more efficient, scalable and intelligent applications of SERS in food safety. For example, conventional machine learning algorithms (such as PCA, LDA, SVM and random forest) are widely employed for feature extraction and dimensionality reduction. Deep learning (such as CNNs, recurrent neural networks and autoencoders) can achieve end-to-end automatic feature learning, noise reduction, spectral information extraction and accuracy enhancement [142]. The integration of these algorithms with detection hardware (e.g., portable Raman spectrometers, microfluidic devices and flexible wearable sensors) facilitates real-time monitoring and intelligent decision-making.

## 5. Conclusions

SERS, with its outstanding performance in terms of its ultra-sensitivity, non-destructiveness and capability for molecular fingerprint has been employed in food contaminant detection. The primary strategies for pollutant detection via SERS can be broadly categorized into two methods: label-free detection and labeled detection. The controllable synthesis of efficient SERS-active nanomaterials is essential for the successful application of SERS. Over time, SERS substrates have evolved from simple noble metal-based structures to more advanced composite materials and polymetallic substrates. Au-Ag BNPs exhibit a synergistic enhancement effect, which results in a stronger SERS signal compared to single-component noble metal nanomaterials. To effectively apply SERS for the rapid detection of harmful pollutants, it is crucial to address challenges related to the stability and functionalization of nanoparticles, interference from sample matrices, and the need for on-site portability. Future research should focus on the development of intelligent, integrated substrates and cost-effective, user-friendly SERS detection platforms. Additionally, integrating cutting-edge technologies such as artificial intelligence and microfluidics will help meet the demands for on-site detection.

## Figures and Tables

**Table 1:** Typical research progress on the detection of food contaminants by SERS technology.

Contaminants	Plasmonic Nanostructures	Detection Method	Extra Technology or Functional Molecules	Limit of Detection	References
*E. coli*	Au@Ag NRs	Labeled	Antibody	10^2^ CFU/mL	[55]
*E. piscicida*, *E. coli*, *V. anguillarum*, *V. harveyi* and *P. plecoglossicida*	Au@Ag NPs	Label-free	Separation and enrichment of magnetic materials	Classification; 10^5^ CFU/mL (*E. piscicida*)	[56]
*S. aureus*	Au-assisted magnetic nanoparticles; Au@Ag-ATP	Labeled	Hybridization chain reaction, aptamer	0.25 CFU/mL	[57]
*E. coli* and *S. aureus*	AuAg@PB MOF	Label-free	4-MPBA-functionalized substrate, dual-modal sensing	42 CFU/mL (*E. coli*); 45 CFU/mL (*S. aureus*)	[58]
*Listeria innocua*	AuAg@pSiNWs	Label-free	/	1.14 × 10^4^ CFU/mL	[59]
*Salmonella*	Au-Ag NPs/Si	Label-free	/	1 CFU/mL	[60]
*Clostridium perfringens* , *Bacillus subtilis* and *S. aureus*	Au@AgNPs/Van-PDMS	Label-free	Machine learning algorithms	3 CFU/mL (*S. aureus*)	[61]
*S. aureus*	Au@AgPt nanozyme array	Label-free (indirect)	Dual mode	38 CFU/mL (colorimetric); 6 CFU/mL (SERS)	[62]
*P. aeruginosa*, *S. typhimurium* and *E. coli*	MoDAu@Ag	Labeled	Antibody, LFIA	29 cells/mL; 34 cells/mL; 40 cells/mL	[63]
*E. coli*, *S. aureus* and *P. aeruginosa*	Au-Ag-stuffed nanopancakes	Labeled (internal standard)	Triple-functional substrates	7 CFU/mL	[64]
PAT	Au@Ag^MBA^ NPs; AuNSs-cDNA	Labeled	Aptamer	0.0281 ng/mL	[65]
ZEN	Au^MBA^@Ag^MBA^ NPs	Labeled	Antibody	3 μg/kg	[23]
ZEN	Au^DTNB^@Ag NPs	Labeled	Aptamer	0.001 ng/mL	[66]
OTA	Au^MBA^@AuAg NPs	Labeled	Aptamer	0.004 ng/mL	[67]
AFB_1_	Au-Ag Janus NPs	Labeled	Aptamer	0.5 pg/mL	[37]
AFB_1_	Au^MBA^@Ag NPs; ITO-Au-GO	Labeled	Aptamer	0.1 pg/mL	[68]
AFB_1_	Au@Ag bimetallic nanostars	Labeled	Aptamer	58.9 pg/mL	[69]
AFB_1_	Ag@Au IP6 bifunctional nanozymes	Labeled	Dual mode, aptamer	0.58 pg/L	[70]
DON	AuNR@Ag@SiO_2_-AuNP	Labeled	Antibody, LFIA	0.053 fg/mL	[71]
AFB_1_ and ZEN	Fe_3_O_4_@PEI/Au^MBA^@Ag^MBA^	Labeled	Antibody, LFIA	0.095 μg/kg (AFB_1_); 1.896 μg/kg (ZEN)	[72]
OTA, AFB_1_ and ZEN	Au^NBA^@Ag, Au^4-MBA^@Ag and Au^DNTB^@Ag	Labeled	Antibody, vertical flow immunoassay	8.2 fg/mL (OTA), 13.7 fg/mL (AFB_1_), 47.6 fg/mL (ZEN)	[73]
PAT	Au@Ag NPs, MOF and AuNRs	Labeled	Aptamer	0.0465 ng/mL	[74]
Thiabendazole	Au@Ag NRs	Label-free	/	0.032 mg/kg	[75]
Thiram and acetamiprid	Au@Ag NPs	Label-free	Simultaneous detection	0.076 μM (thiram); 1.22 μM (acetamiprid)	[11]
2,4-D and imidacloprid	Au@Ag nanoflowers	Label-free	Chemometric algorithms	2.98 μg/L (2,4-D); 5.5 μg/L (imidacloprid)	[76]
Thiram	PAN/Cu_2_O@Ag/Au@AgNPs	Label-free	Deep learning algorithm	0.02 mg/L	[77]
Thiram and thiabendazole	Au@4-MBA@Ag array	Label-free	Ratio Raman	0.38 μg/L (thiram); 25 μg/L (thiabendazole)	[78]
Acetamiprid and carbendazim	Au^MBA^@Ag^MBA^ NPs	Labeled	LFIA	0.27 μg/kg (acetamiprid); 1.71 μg/kg (carbendazim)	[79]
Crystal violet, thiram and carbaryl	SiO_2_@AuAg	Label-free	/	6.95 × 10^−7^ M (crystal violet); 5.56 × 10^−7^ M (thiram); 7.14 × 10^−6^ M (carbaryl)	[80]
Thiram and pymetrozine	Au-Ag octahedral hollow cages	Label-free	Machine learning algorithms	0.286 μg/kg (thiram); 29 μg/kg (pymetrozine)	[34]
Thiram	Cellulose nanofiber/AuNRs@Ag	Label-free	/	10^−11^ M	[81]
Malachite green	Chiral spiny L-Au@Ag@ZIF-8	Label-free	/	6.56 × 10^−10^ M	[82]
Acetamiprid	Ag@ZIF-8@Au nanoparticles	Label-free	/	9.027 × 10^−10^ M	[83]
Carbaryl	ZIF-8@Ag/AAB/Au@Ag	Label-free	/	5.72 × 10^−3^ µg/mL	[84]
Chloramphenicol	Ascorbate-functionalized Au@Ag NPs	Label-free	Chemometric algorithms	2.73 × 10^−5^ μg/mL	[85]
Chloramphenicol	Au@Ag NBPs/SiO_2_ nanoarray	Labeled	DNA enzyme amplification strategy	6.42 × 10^−13^ M	[86]
Amoxicillin and fenobucarb	Ag-Au alloy nanoparticles	Label-free	/	/	[87]
RhB	Ag@SiO_2_-Au NPs	Label-free	/	5 × 10^−9^ M	[88]
Paracetamol and furazolidone	Ag/Au/AgCl heterostructure	Label-free	/	2.8 × 10^−12^ M (paracetamol); 1.9 × 10^−11^ M (furazolidone)	[89]
Ciprofloxacin and chloramphenicol	TiO_2_/Au/Ag nanorod arrays	Label-free	/	10^−9^ M (ciprofloxacin); 10^−8^ M (chloramphenicol)	[90]
R6G	WS_2_/Au@Ag nanocomposites	Label-free	Deep learning algorithm	10^−14^ M	[91]
Chloramphenicol	Au@Ag NPs	Label-free	chemometric algorithms	1 × 10^−5^ μg/mL	[92]
Tetracycline	Fe_3_O_4_@h-TiO_2_/Au nanochains and Au@Ag NPs	Labeled	Aptamer, cascade amplification	15.91 pg/mL	[93]
Cr (VI)	Au@Ag nano-sea urchins	Labeled	Methimazole-functionalized	0.956 ng/L	[94]
Pb^2+^	Au@Ag NRs	Labeled	Glutathione and 4-MBA-functionalized	0.021 μg/L	[95]
Hg^2^^+^	Au@Ag NPs	Labeled	Colorimetric/SERS dual-mode, etching Ag shell	2 μM (naked eye); 0.2 nM(UV-vis); 0.1 nM (SERS)	[96]
Hg^2^^+^	Au@Ag/graphene-upconversion nanohybrids	Labeled	Fluorescence/SERS dual-mode, aptamer	0.33 ppb (SERS); 1 ppb (fluorescence)	[97]
Hg^2^^+^	Au@Ag/COF	Labeled	Y-shaped DNA-functionalized	5.0 × 10^−16^ M	[98]
R6G, thiram, melamine and piroxicam	Au@SiO_2_@Ag@SiO_2_ composites	Label-free	/	10^−9^ M (R6G); 10^−6^ M (thiram); 10^−3^ M (melamine); 10^−3^ M (piroxicam);	[99]
Bacteria spores	Ag@AuNP array	Label-free	Chemometric algorithms	10 CFU/mL	[100]
Malachite green	Au nanobipyramid@Ag	Label-free	/	0.1 nM	[101]
Amaranth and allura red	PDDA/Ag/Au hybrid plasmonic optical cavity	Label-free	/	0.3022 mg/L (amaranth); 0.2482 mg/L (allura red)	[102]
Six colorants	GO/Au@Ag nanobones	Label-free	Machine learning algorithms	/	[103]
Polystyrene nanoplastics	Ag@Au film	Label-free	/	25 (310 nm), 50 (50, 70 nm) μg/mL	[104]
SEC	Au-Ag Janus NPs	Labeled	MBIA-functionalized, antibody	0.55 pg/mL	[105]
SEC	Au-Ag Janus NPs	Labeled	MBIA-functionalized, aptamer	0.83 pg/mL	[106]
β-lactoglobulin	Au-Ag nanourchins	Labeled	Aptamer	0.07 ng/mL	[107]

*E. coli*, *Escherichia coli*; *E. piscicida*, *Edwardsiella piscicida*; *V. anguillarum*, *Vibrio anguillarum*; *V. harveyi*, *Vibrio harveyi*; *P. plecoglossicida*, *Pseudomonas plecoglossicida*; *S. aureus*, *Staphylococcus aureus*; AuAg@PB MOF, AuAg-doping Prussian blue analogue-based metal–organic framework; 4-MPBA, 4-mercaptophenylboronic acid; AuAg@pSiNWs, gold- and silver-coated porous silicon nanowires; *P. aeruginosa*, *Pseudomonas aeruginosa*; *S. typhimurium*, *Salmonella typhimurium*; 2,4-D, 2,4-dichlorophenoxyacetic acid; PAN, polyacrylonitrile; LFIA, lateral flow immunoassay; PAT, patulin; ZEN, zearalenone; OTA, ochratoxin A; AFB_1_, Aflatoxin B1. DON, deoxynivalenol; AAB, artificial antibody; COF, covalent organic framework; PDDA, poly(diallyldimethylammonium chloride); GO, graphene oxide; SEC, *Staphylococcus aureus* enterotoxin C; MBIA, 2-mercaptobenzoimidazole-5-carboxylic acid.

## Data Availability

The original contributions presented in the study are included in the article; further inquiries can be directed to the corresponding author.
